# Commentary: Craving in Opioid Use Disorder: From Neurobiology to Clinical Practice

**DOI:** 10.3389/fpsyt.2021.615921

**Published:** 2021-08-09

**Authors:** Rui Zheng, Liu Hao, Yuanhui Li, Tianjiao Zhang, Dexiang Bai, Liqun Zhang, Dai Li, Wei Hao

**Affiliations:** ^1^Adai Technology (Beijing) Ltd., Co, Beijing, China; ^2^Department of Clinical Laboratory, Second Xiangya Hospital, Central South University, Changsha, China; ^3^Mental Health Institute of the Second Xiangya Hospital, National Clinical Research Center on Mental Disorders Central South University, Changsha, China

**Keywords:** craving, objectively assessing, virtual reality, machine learning, biomarker

## Introduction

We read with great interest the review article by Kakko et al. (1), which focused on the opioid use disorder-related craving. A recent review also discussed underlying neurobiological changes in opioid use disorder (OUD) that likely contribute to drug craving (2). However, how to assess the craving in clinical practice is challenging. Here, we would like to present the recent developments and briefly introduce our work on objectively assessing craving.

### Current Practice of Evaluating Drug Addiction Cravings and the Limitations

Craving is the central concept of almost all major drug dependence models (3–7). In the International Classification of Diseases [ICD-11 (8)], craving is listed as one of the six characteristics of psychoactive substance dependence. In DSM-5 (9, 10), craving has once again become the recommended standard for diagnosing substance use disorder (SUD).

Current craving assessments are often accomplished through self-reports and observations of cognitive performance (11).

Despite the development of several psychometrically validated multi-item craving inventory tools (12, 13), self-reported cravings are still most commonly evaluated using a single item craving inventory (11). Since the intensity, latency, frequency, and significance of craving episodes may vary (14), self-reported assessments may have difficulty in reflecting true differences of cravings among patients (15). In daily clinical experiences, denial is one of the typical responses when we ask patients with alcohol and drug problems for their addictive behaviors, and in certain situations, for instance, in order to gain acceptance from family members, or gain enough trust to seek drugs privately, patients may conceal the true level of cravings.

Cognitive performance tasks are used to implicitly assess cravings (6, 16), based on the assumption that craving leads to the redistribution of cognitive processing resources and shifts that to drug-related cues (6). Therefore, facing the drug-related cues at a certain intensity, people with different intensities of drug addiction cravings would show different cognitive performances. Through recording cognitive performance parameters (e.g., the reaction time), it could reflect the situations of redistribution of cognitive processing resources, and therefore indirectly reflect the related craving. This indirect assessment method may avoid subjective bias (15) and may help to understand how craving changes perceptions and decisions. However, it is very challenging to implement in clinical practice (11, 12).

Kakko et al. (1) also mentioned using ecological momentary assessments (EMA) to assess cravings (17, 18). EMA uses mobile technology to record a real-time (including daily, random, or event-triggered) cravings (17–20). However, EMA only improves the understanding of craving-related time fluctuations (17, 18), but fail to improve craving assessment itself.

### Current Approach of the Assessment of Induced Cravings and the Limitations

In addition to Background cravings (7), cravings can also be induced through specific drug-related prompts or stressful life events (7). Most craving-inducing studies use the cue-reactivity paradigm (7), in which drug users respond differently to drug-related cues, in contrast with neutral cues, resulting in changes in brain activity related to the degree of craving (7).

The way to induce cravings at present depends mainly on the contents of pictures or videos. Although related to drug-seeking behavior and successfully induced cravings, they may not trigger reactions representing the real social or environmental cues (21), of which studies have shown that the treatment requires intervention (21).

### New Approach to Evaluating Drug Addiction Cravings With New Technology

Our research team has designed the VR paradigm (22, 23) to improve the way to induce cravings, which can simulate the real world with the implicit cue, rebuild specific environments and characters, and allow certain social immersion and interaction, as well as the balanced drug-related cue and neutral stimuli. By technically arranging the sequence of stimuli and social interactions in the scenes, an effective evaluation experimental paradigm is formed.

Our results showed that the effects of different cues are related to the types of drug dependence, and VR stimulation could cause a higher level of cravings than the traditional picture and video stimulations, consistent with previous findings (14, 24). Through different types of induced stimuli, specific incentives for craving can provide a valuable therapeutic reference for clinical consultation and treatment.

During VR stimulation, we recorded several physiological parameters, including electroencephalogram (EEG), galvanic skin response, and heart rate (22, 23). We employed machine learning methods to analyze these physiological data to distinguish drug-dependent individuals from normal controls (22, 23).

We used machine learning algorithms, including random forest, AdaBoost, logistic regression, and ensemble model to build classification models (22, 23). The most striking findings were that the highest classification accuracy of 86% was obtained by the AdaBoost algorithm, with 84% precision, 83% recall rate, and 83% f1 score (23). Our results show that compared with traditional statistical methods, machine learning has advantages in processing massive and complex physiological data, which helps identify features that can distinguish different populations.

## Discussion

The level of craving is unstable and affected by multiple factors and changes along with the internal and external environments over time, while the patient's dependent state is relatively stable. Thus, it is possible to find stable biomarkers reflecting a patient's status related to dependence rather than reflecting the real-time craving level. This is urgently needed for clinical practice, not only for objective diagnosis and severity of dependence but also to be used as an early sign for relapse.

Because of the complexity of mental illnesses such as addiction, the traditional research methods based on reductionism were limited and overwhelmed (25). The emergence of new technologies, such as machine learning, has made holistic research possible in this field. The previous reductionist studies are hypothesis-driven; that is, they explore the regularity based on a relatively clear hypothesis. Such studies often focus on specific mechanisms, and the results are highly explanatory, while machine learning studies are driven by holistic data. Through in-depth mining of overall data, machine learning studies could provide more detailed results and new directions, although the interpretability is sometimes poor.

The advantage of the holism method should be considered with the help of multidisciplinary forces and high-level technology, such as biophysics, non-linear computing, and machine learning. Using holism methods to find the regularity first and then exploring the specific mechanism may be a promising solution. We developed a model that could accurately distinguish drug-dependent individuals and normal controls by using machine learning algorithms, obtained potential key biomarkers of the dependent state, and achieved some promising results (26).

At the same time, we explored the correlation between the probability results of machine learning algorithms and some other clinical indicators, including not only the subjective craving inventory score but also drug use-related indicators, such as drug use duration, dosage, and frequency. Since the correlation could be intricate, we track and analyze most of the relevant indicators. Even though the research may not yield satisfying results in the short term, using machine learning to classify the population is meaningful in itself and provides a direction for further exploration of the biological nature of dependence and addiction.

## Author Contributions

WH initiated the project and designed the work. RZ and LH were approached to write the paper, structured it, and wrote sections Introduction and Current Practice of Evaluating Drug Addiction Cravings and the Limitations. YL and TZ wrote sections Current Approach of the Assessment of Induced cravings and the Limitations and New Approach to Evaluating Drug Addiction Cravings With New Technology. DB and LZ wrote section Discussion. RZ, LH, DL, and WH revised the manuscript. All authors contributed to the article and approved the submitted version.

## Conflict of Interest

RZ, YL, TZ, DB, LZ, and DL were employed by the company Adai Technology (Beijing) Ltd., Co. The remaining author declares that the research was conducted in the absence of any commercial or financial relationships that could be construed as a potential conflict of interest.

## Publisher's Note

All claims expressed in this article are solely those of the authors and do not necessarily represent those of their affiliated organizations, or those of the publisher, the editors and the reviewers. Any product that may be evaluated in this article, or claim that may be made by its manufacturer, is not guaranteed or endorsed by the publisher.

## References

[1] Kakko J Alho H Baldacchino A Molina R Nava FA Shaya G. Craving in opioid use disorder: from neurobiology to clinical practice. Front Psychiatry. (2019) 10:592. 10.3389/fpsyt.2019.00592 31543832 PMC6728888

[2] Lueptow LM Shashkova EC Miller MG Evans CJ Cahill CM. Insights into the neurobiology of craving in opioid use disorder. Curr Anesthesiol Rep. (2020) 10:378–387. 10.1007/s40140-020-00420-7 33424457 PMC7790122

[3] Drummond DC. Theories of drug craving, ancient and modern. Addiction. (2001) 96:33–46. 10.1046/j.1360-0443.2001.961333.x 11177518

[4] Tiffany ST Warthen MW Goedeker KC. The functional significance of craving in nicotine dependence. Nebr Symp Motiv. (2009) 55:171–97. 10.1007/978-0-387-78748-0_10 19013944

[5] Baker TB Piper ME McCarthy DE Majeskie MR Fiore MC. Addiction motivation reformulated: an affective processing model of negative reinforcement. Psychol Rev. (2004) 111:33–51. 10.1037/0033-295X.111.1.33 14756584

[6] Tiffany ST. A cognitive model of drug urges and drug-use behavior: role of automatic and nonautomatic processes. Psychol Rev. (1990) 97:147–68. 10.1037/0033-295X.97.2.147 2186423

[7] Tiffany ST Wray JM. The clinical significance of drug craving. Ann N Y Acad Sci. (2012) 1248:1–17. 10.1111/j.1749-6632.2011.06298.x 22172057 PMC4041083

[8] ICD-11: International Classification of Diseases 11th Revision. Geneva: World Health Organization (2018). Available online at: https://icd.who.int/browse11

[9] American Psychiatric Association Task Force On DSM-V. Diagnostic and Statistical Manual of Mental Disorders: DSM-V. 5th ed. (2013). 10.1176/appi.books.9780890425596

[10] Hasin DS O'Brien CP Auriacombe M Borges G Bucholz K Budney A. DSM-5 criteria for substance use disorders: recommendations and rationale. Am J Psychiatry. (2013) 170:834–51. 10.1176/appi.ajp.2013.12060782 23903334 PMC3767415

[11] Rosenberg H. Clinical and laboratory assessment of the subjective experience of drug craving. Clin Psychol Rev. (2009) 29:519–34. 10.1016/j.cpr.2009.06.002 19577831

[12] Sayette MA Shiffman S Tiffany ST Niaura RS Martin CS Shadel WG. The measurement of drug craving. Addiction. (2000) 95(Suppl. 2):S189–210. 10.1046/j.1360-0443.95.8s2.8.x 11002914 PMC2683662

[13] McHugh RK Fitzmaurice GM Carroll KM Griffin ML Hill KP Wasan AD. Assessing craving and its relationship to subsequent prescription opioid use among treatment-seeking prescription opioid dependent patients. Drug Alcohol Depend. (2014) 145:121–6. 10.1016/j.drugalcdep.2014.10.002 25454409 PMC4254575

[14] Hone-Blanchet A Wensing T Fecteau S. The use of virtual reality in craving assessment and cue-exposure therapy in substance use disorders. Front Hum Neurosci. (2014) 8:844. 10.3389/fnhum.2014.00844 25368571 PMC4201090

[15] Sayette MA. The role of craving in substance use disorders: theoretical and methodological issues. Annu Rev Clin Psychol. (2016) 12:407–33. 10.1146/annurev-clinpsy-021815-093351 26565121

[16] Field M Munafo MR Franken IH. A meta-analytic investigation of the relationship between attentional bias and subjective craving in substance abuse. Psychol Bull. (2009) 135:589–607. 10.1037/a0015843 19586163 PMC2999821

[17] Serre F Fatseas M Swendsen J Auriacombe M. Ecological momentary assessment in the investigation of craving and substance use in daily life: a systematic review. Drug Alcohol Depend. (2015) 148:1–20. 10.1016/j.drugalcdep.2014.12.024 25637078

[18] Huhn AS Harris J Cleveland HH Lydon DM Stankoski D Cleveland MJ. Ecological momentary assessment of affect and craving in patients in treatment for prescription opioid dependence. Brain Res Bull. (2016) 123:94–101. 10.1016/j.brainresbull.2016.01.012 26876756 PMC4919812

[19] Moskowitz DS Young SN. Ecological momentary assessment: what it is and why it is a method of the future in clinical psychopharmacology. J Psychiatry Neurosci. (2006) 31:13–20. 16496031 PMC1325062

[20] Preston KL Jobes ML Phillips KA Epstein DH. Real-time assessment of alcohol drinking and drug use in opioid-dependent polydrug users. Behav Pharmacol. (2016) 27:579–84. 10.1097/FBP.0000000000000250 27579810 PMC5010032

[21] Perry JL Joseph JE Jiang Y Zimmerman RS Kelly TH Darna M. Prefrontal cortex and drug abuse vulnerability: translation to prevention and treatment interventions. Brain Res Rev. (2011) 65:124–49. 10.1016/j.brainresrev.2010.09.001 20837060 PMC3005953

[22] Ding X Li Y Gong L Li D Peng Z Bi Y. A machine learning approach to detect methamphetamine craving induced in a virtual reality environment. In: AAAP 29th Annual Meeting and Scientific Symposium. Bonita Springs, FL (2018).

[23] Ding X Li Y Qi Y Bi Y Tang H Li D. A machine learning approach using EEG data to detect methamphetamine craving induced in a virtual reality environment. In: Society for Neuroscience 2018-Behavioral Studies of Amphetamines. San Diego, CA (2018).

[24] McHugh RK Fulciniti F Mashhoon Y Weiss RD. Cue-induced craving to paraphernalia and drug images in opioid dependence. Am J Addict. (2016) 25:105–9. 10.1111/ajad.12344 26848719 PMC4883011

[25] Choudhury S Slaby J. Critical Neuroscience: A Handbook of the Social and Cultural Contexts of Neuroscience. Re-Socializing Psychiatry. (2011). p. 305–30. 10.1002/9781444343359.ch15

[26] Ding X Li Y Li D Li CL Liu X^*^. Using machine learning approach to distinguish patients with methamphetamine dependence from healthy subjects in a virtual reality environment. Brain Behav. (2020) 10:e01814. 10.1002/brb3.1814 32862513 PMC7667292

